# Evaluation of the Number of Degrees of Freedom of the Field Scattered by a 3D Geometry

**DOI:** 10.3390/s23084056

**Published:** 2023-04-17

**Authors:** Ehsan Akbari Sekehravani, Giovanni Leone, Rocco Pierri

**Affiliations:** Department of Engineering, University of Campania “Luigi Vanvitelli”, I-81031 Aversa, Italy; ehsan.akbarisekehravani@unicampania.it (E.A.S.); rocco.pierri@unicampania.it (R.P.)

**Keywords:** linear inverse scattering, number of degrees of freedom, 3D geometry, microwave tomography of dielectric object, TSVD inversion

## Abstract

The solution to an ill-posed linear inverse problem requires the use of regularization methods to achieve a stable approximation solution. One powerful approach is the truncated singular value decomposition (TSVD), but it requires an appropriate choice of the truncation level. One suitable option is to take into account the number of degrees of freedom (NDF) of the scattered field, which is defined by the step-like behavior of the singular values of the relevant operator. Then, the NDF can be estimated as the number of singular values preceding the knee or the exponential decay. Therefore, an analytical estimation of the NDF is significant for obtaining a stable, regularized solution. This paper addresses the analytical estimation of the NDF of the field scattered by the surface of a cube geometry for a single frequency and the multi-view case in the far-zone. In addition, a method is proposed to find the minimum numbers of plane waves and their directions to achieve the total estimated NDF. The main results are that the NDF is related to the measure of the surface of the cube and can be achieved by only considering a limited number of impinging plane waves. The efficiency of the theoretical discussion is demonstrated through a reconstruction application for microwave tomography of a dielectric object. Numerical examples are provided to confirm the theoretical results.

## 1. Introduction

Inverse problems are not only important from a mathematical point of view, but also because they arise in many engineering applications. In general, when the output and the input/system are known, then the task is to compute the system/input. An inverse problem is well-posed if it satisfies the three requirements of Hadamard [1], otherwise, the problem is ill-posed. In particular, it is not possible to compute the solution stably because a minor perturbation of data (due to noise or uncertainties) can yield a wide perturbation of the solution. It is required to stabilize the solution using a suitable regularization method.

The influence of noise on data can be filtered out by regularization of the problem, such as Tikhonov regularization [2] and the truncated singular value decomposition (TSVD) [3]. These regularization techniques rely on a regularization parameter that determines how much filtering is added as a result of the regularization. The major challenge of these methods is finding a suitable regularization parameter that provides sufficient filtering noise out without losing too much information in the regularized solution.

Indeed, a good regularization parameter in the TSVD method is the so-called number of degrees of freedom(NDF), which is the number of independent pieces of information that can be reconstructed stably in the presence of noise on data. The NDF of a compact linear operator can be estimated as the number of significant singular values when the singular values exhibit a step-like behavior. Unfortunately, the NDF can only be found in closed form only for some circumstances. The NDF concept has been studied in electromagnetics problems in [4,5,6]. For instance, the NDF evaluation has been addressed for strip geometries [7] and circumference geometry [8] in inverse source problems and in linear inverse scattering problems for strip [9] and circumference [8] geometries. The results of [10] have shown that the NDF of the 2D scattering object depends on its area and the area of the angular domain of incident plane waves and observations. Sometimes, the NDF can be only computed numerically, as in [11] for the NDF of the field scattered by curve geometry for aspect-limited observations and excitations.

The concept of NDF of the field has been utilized in various applied electromagnetic fields [12], such as in inverse source problem [13], multiple input multiple output (MIMO) channel [14,15], MIMO radar and imaging [16], and microwave tomography [5]. Additionally, it has been used in antenna measurements [17], 3D linear large-scale antenna array communications [18], and radar cross section evaluation [19]. In [20], the usefulness of determining the number of independent communication modes in optical communications and, in general, in communication channels, taking into account the scattering environment [21,22], has also been proven. Therefore, it can be concluded that it provides a theoretical tool to analyze the performance of a scattering environment, and at the same time, it describes the achievable complexity over the investigation’s domain, affecting the imaging performances. 

The NDF can also be assumed to be the number of point-like scatterers that can be accurately reconstructed for a localization problem, making it significantly important in solving inverse scattering problems. Its estimation requires some a priori information, such as the geometry and size of the scattering object and its location.

Another point of investigation of the paper concerns the minimum number of impinging plane waves required to achieve the total estimated NDF. This information is also useful for reducing the size of the scattering matrix and obtaining a good reconstruction at the same time. This topic has been explored in [8,9,23] for other different simple geometries.

In this paper, we address the evaluation of the NDF of far-field scattered by a three-dimensional (3D) geometry for a single frequency and multi-view/multi-static case in the far-zone, extending our previous NDF results for simpler geometries. To this end, we first recall the NDF of the volume of a cube, then investigate the surface of a cube as investigation domain. Solving this kind of problem is of interest in real applications and is preliminary for considering general surface investigation domains. We start with the case of a planar surface, then analyze the collection of two sides of the cube. In addition, we propose a method for choosing the optimal number of impinging plane waves and their directions to achieve the total estimated NDF. Finally, we introduce an application to highlight the efficiency of the theoretical discussion in reconstruction. To the authors’ knowledge, this is the first time that an analytical evaluation has been made possible in a 3D geometry.

This paper is organized as follows: Section 2 is devoted to presenting the statement of the problem. The steps for evaluating the NDF of the surface of a cube are provided in Section 3. Section 4 discusses how to find the optimal number of plane waves and their directions. A numerical application of the theoretical discussions is provided in Section 5. The conclusions follow in Section 6.

## 2. The Statement of the Problem

**Figure 1:**
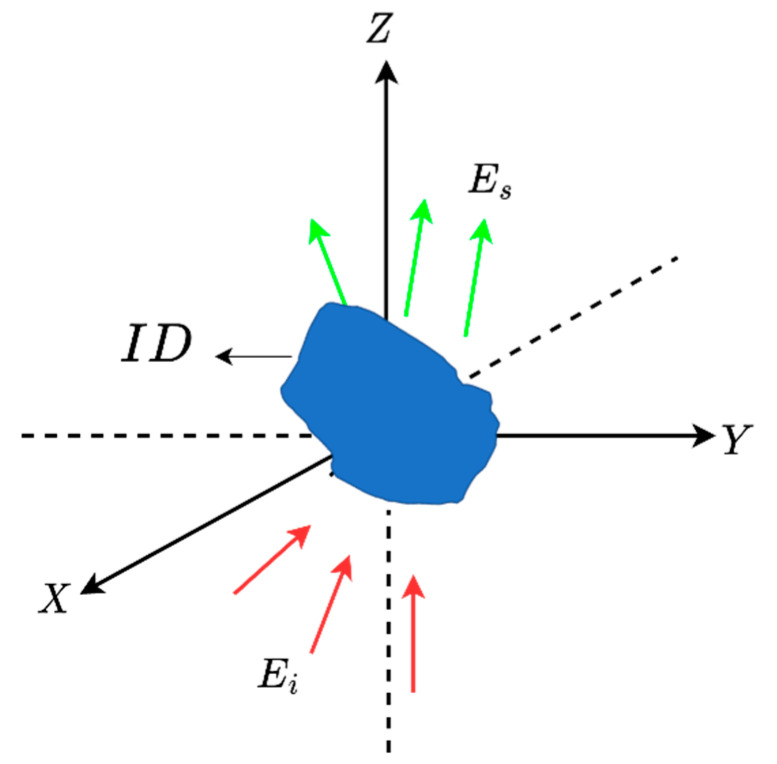
The geometry of the problem.

The inverse scattering problem is formulated for a general 3D scatterer geometry, as shown in Figure 1. An unknown scatterer is located in a 3D domain referred to as Investigation Domain (ID). It is supposed that the scatterer is situated in a homogeneous medium with dielectric permittivity $\varepsilon_{0}$ and magnetic permeability $\mu_{0}$. The angles of incident plane waves are defined by $\theta_{i}\in \left(0,\pi \right)$ and $\phi_{i}\in \left(0,2\pi \right)$, which indicate their direction of propagation. Meanwhile, the observation angles of the scattered far field are represented by $\theta_{s}\in \left(0,\pi \right)$ and $\phi_{s}\in \left(0,2\pi \right)$, respectively.

The scattered field in the far-zone under the Born approximation for the 3D scalar case is given by:

(1) $$E_{s}(\theta_{s},\theta_{i},\phi_{s},\phi_{i})\;\;\;\;\;\;\;\;\;\;\;\;\;\;\;\;\;\;\;\;\;\;\;\;\;\;\;\;\;\;={\int \int \int }_{ID}\chi \left(x,y,z\right)e^{j\beta \left[x\left(\sin\theta_{s}\cos\phi_{s}-\sin\theta_{i}\cos\phi_{i}\right)+y\left(\sin\theta_{s}\sin\phi_{s}-\sin\theta_{i}\sin\phi_{i}\right)+z\left(\cos\theta_{s}-\cos\theta_{i}\right)\right]}\;dx\;dy\;dz=\;\;\;T\left(\chi \left(x,y,z\right)\right)\;\;\;\;\;\;\;\;\;\;\;\;\;\;\;\;\;\;\;\;\;\;\;\;\;\;\;\;\;$$

where T is the linear operator for the multi-view and single frequency, and χ(x,y,z) is the contrast function. The scatterer with a relative permittivity of $\epsilon_{s}\left(x,y,z\right)$, therefore, $\chi \left(x,y,z\right)=\left(1-\epsilon_{s}\left(x,y,z\right)\right)/\epsilon_{0}$ is placed in a homogeneous background, which has the permittivity of free space ($\epsilon_{0}=8.854\times 10^{-12}\;F/m$). The wavenumber β is represented as $\beta =\omega \sqrt{\left(\epsilon_{0}\mu_{0}\;\right)}=2\pi /\lambda$, where ω is the angular frequency, $\mu_{0}=4\pi \times 10^{-7}\;H/m$ is the magnetic permeability of the free space, and λ is the wavelength. 

As T is linear and compact, the SVD approach can be introduced and applied to estimate the NDF as the number of significant singular values. The SVD is comprised of three components: $\left\{v_{n},\sigma_{n},u_{n}\right\}$ [3], where $v_{n}$ and $u_{n}$ represent singular functions, and $\sigma_{n}$ refers to the n-th singular value. The spectral $k_{x}$, $k_{y}$, and $k_{z}$ variables are introduced and employed to estimate the NDF of (1) as:

(2) $$\{\begin{array}{c} k_{x}=\beta \left(\sin\theta_{s}\cos\phi_{s}-\sin\theta_{i}\cos\phi_{i}\right)\\ k_{y}=\beta \left(\sin\theta_{s}\sin\phi_{s}-\sin\theta_{i}\sin\phi_{i}\right)\\ k_{z}=\beta \left(\cos\theta_{s}-\cos\theta_{i}\right) \end{array}$$

**Figure 2:**
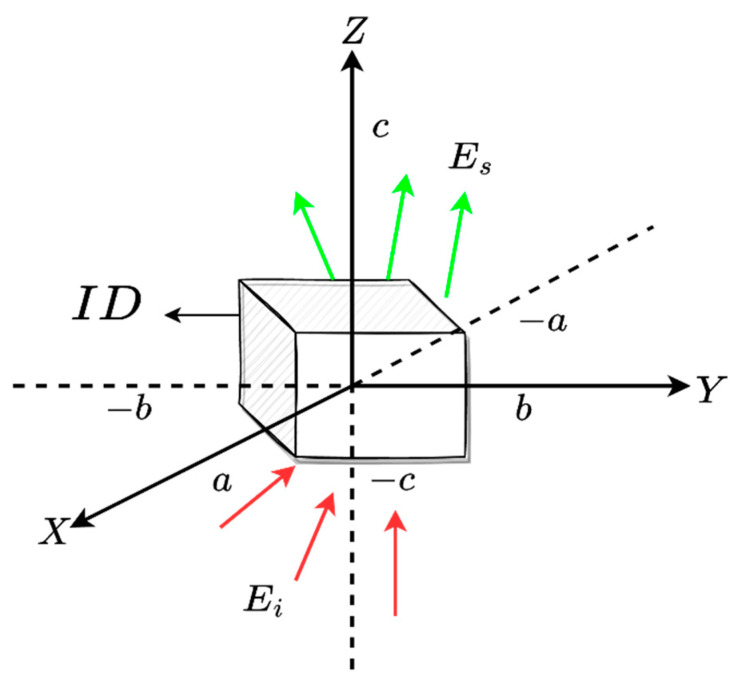
The geometry of a cube ID.

Therefore, (2) reads as:

(3) $$E_{s}\left(k_{x},k_{y},k_{z}\right)={\int \int \int }_{ID}\chi \left(x,y,z\right)e^{j\left(xk_{x}+yk_{y}+zk_{z}\right)}\;dx\;dy\;dz$$

as a triple Fourier transform relationship. We now recall the NDF result from [24] for a general Fourier transform operator, which is given by $NDF=A_{s}\;A_{ID}$, where $A_{s}\in \left(k_{x},k_{y},k_{z}\right)$, $A_{s}$ and $A_{ID}$ are the measure of the volume spectral domain, and the measure of the ID volume, respectively. In addition, $A_{s}$ is determined by the available directions of the incident plane waves and the observation angles of the scattered field. A sphere with a radius of 2β is spanned in the case of full solid angle incidence directions and observations. For instance, the NDF of a cube ID (as shown in Figure 2) is equal to $(4\pi /3){\left(4a/\lambda \right)}^{3}$, if a is its side.

## 3. NDF Evaluation for the Surface of a Cube

While a closed form result of the NDF of the scattered fields is available for a general volume 3D ID, as seen in the previous section, a similar result seems not available for a general surface ID. Therefore, it is worth investigating this point for different surface geometries, in particular, a parallelepiped is considered, as shown in Figure 2. To reach this goal, the full geometry will be built step by step and the corresponding NDF is evaluated at each step.

**Figure 3:**
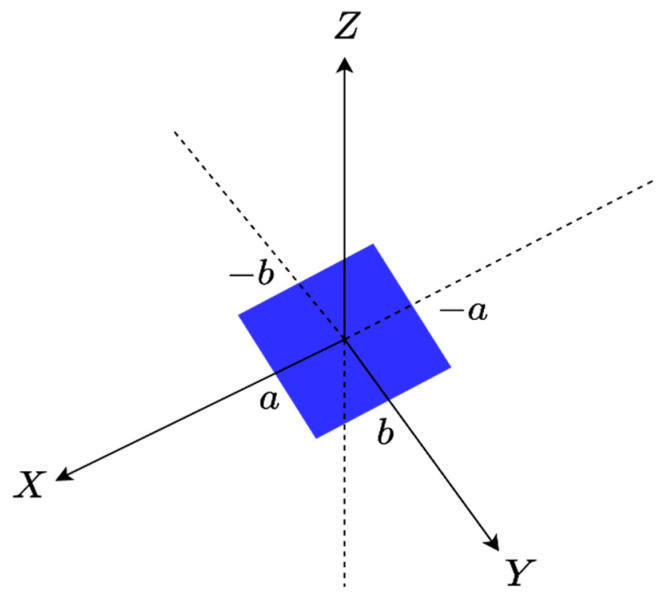
The geometry of the XY face ID.

The first step consists of considering an individual face of the parallelepiped, say one along the XY plane within the 3D spectral domain, when the z variable is constant (z=±c), as shown in Figure 3. Only $k_{x}$ and $k_{y}$ have sense which means that the 3D spectral domain shrinks to a 2D one. Consequently, its scattered field is defined by the 2D Fourier transform relationship.

(4) $$E_{s}\left(k_{x},k_{y}\right)=e^{\pm j\beta ck_{z}}\;\int_{-b}^{b}\int_{-a}^{a}\chi \left(x,y\right)e^{j\left(xk_{x}+yk_{y}\right)}\;dx\;dy$$

In this case, if $k_{z}=0$ in (4), the 3D spectral domain shrinks to 2D as only $k_{x}$ and $k_{y}$ exist. For each $\theta_{i}$ and $\phi_{i}$ incident directions, as $\theta_{s}$ varies, the circle of equation ${\left(\;k_{x}+\beta \sin\theta_{i}\cos\phi_{i}\right)}^{2}+{\left(\;k_{x}+\beta \;\sin\theta_{i}\sin\phi_{i}\right)}^{2}={\left(\beta \sin\theta_{s}\right)}^{2}$ is spanned when considering the multiple numbers of observation directions. When $\left(\theta_{i},\phi_{i}\right)$ vary continuously, the circle with a radius of 2β is filled entirely in the $\left(k_{x},\;k_{y}\right)$ spectral domain.

**Figure 4:**
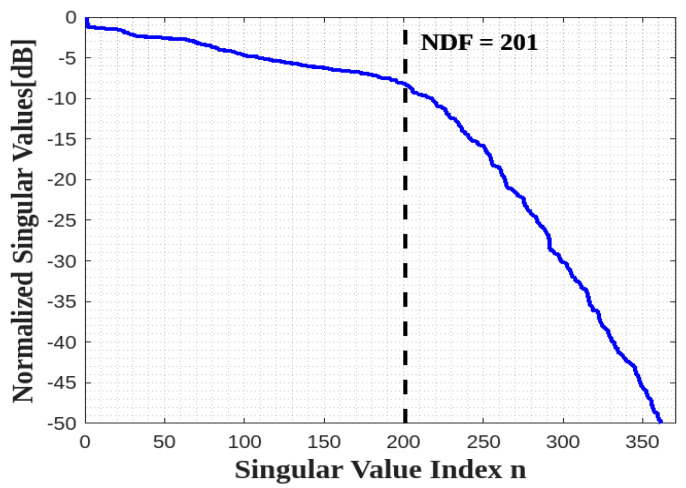
The behavior of normalized singular values for one XY face.

Thus, its NDF is the same as the NDF of a full 2D rectangle in the 2D domain, i.e., $NDF=16ab\pi /\lambda^{2}$ recalled from [10]. Since the NDF are related to the Singular Values (SVs) of (1) for an ID, which is composed of planar surfaces, we adopt the moment method to numerically evaluate the pertinent SVD. In particular, we employ the 2D rectangular pulse functions to approximate the unknown and the Dirac functions to test the resulting approximated integral. When a sufficiently adequate discretization is adopted the SVD of the resulting matrix equation under the MATLAB environment provides the Singular Values of (1) with good accuracy. Figure 4 shows the behavior of normalized SVs of (4) for a=b=2λ and c=0. Thus, the numerical result verifies the NDF analytical estimation. Incidentally, the above discussion is not restricted to a rectangular ID but holds for any planar 2D shape.

**Figure 5:**
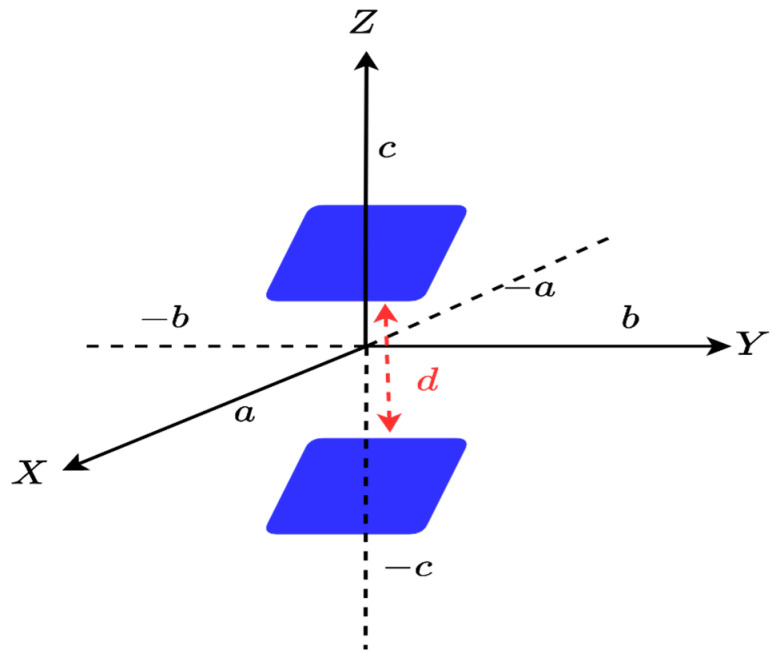
The geometry of two parallel XY faces.

Then, the previous step is extended to consider two parallel XY faces, as shown in Figure 5, and d is the distance between two faces. The approach used in [7,9], which introduced a matrix operator (see Appendix A for more details) and analyzed the significant singular values, which showed that when the distance between two scatterers is not too small, their total NDF can be the same as the sum of their individual NDFs. Therefore, if we consider to compute the NDF of two faces here, it can be expected that the total number of NDF will be $NDF=32ab\pi ⁄\lambda^{2}$.

**Figure 6:**
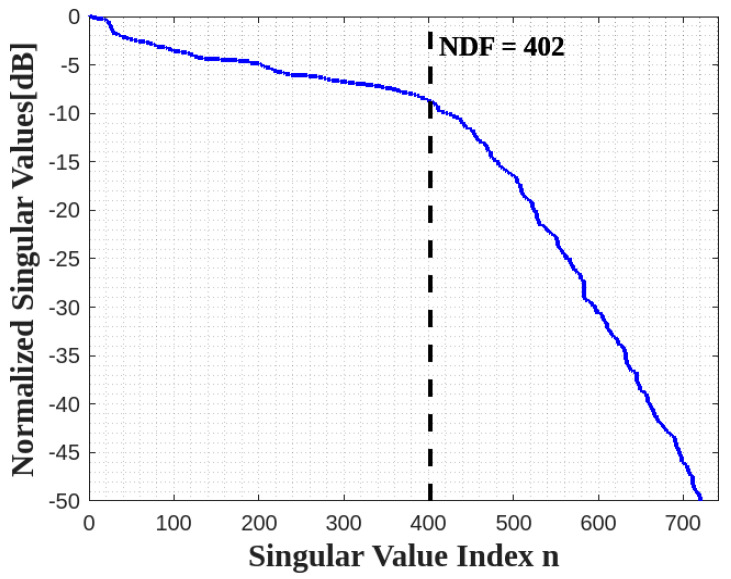
The behavior of normalized singular values for two parallel XY faces.

The behavior of normalized singular values for the two faces ID is plotted in Figure 6 for a=b=2λ, c=±2λ, and it confirms the expectation, i.e., the NDF of two faces is equal to the summation of the NDF of each face. 

**Figure 7:**
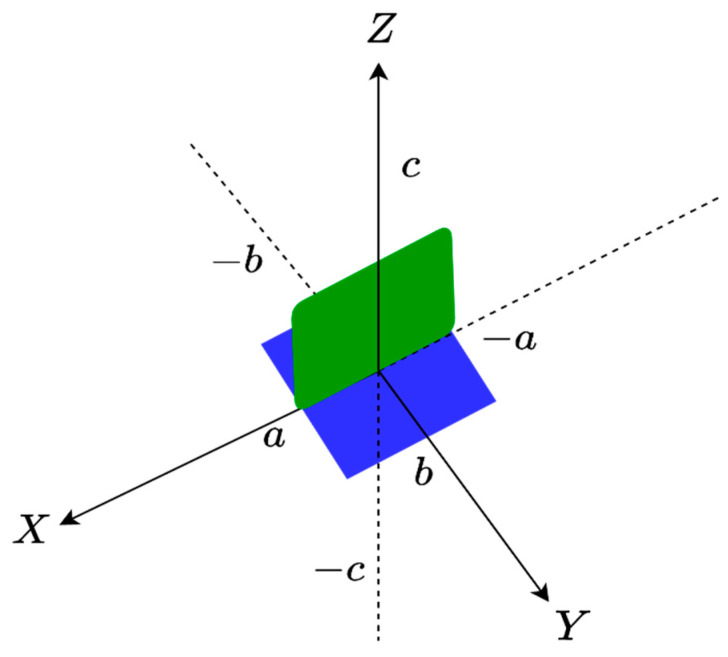
The geometry of the XY and XZ faces for an L-shaped ID.

In the following step, an ID composed of one XY face with one XZ face as an L shape is considered, as shown in Figure 7, to which we can apply the same approach as before in order to compute the total NDF.

**Figure 8:**
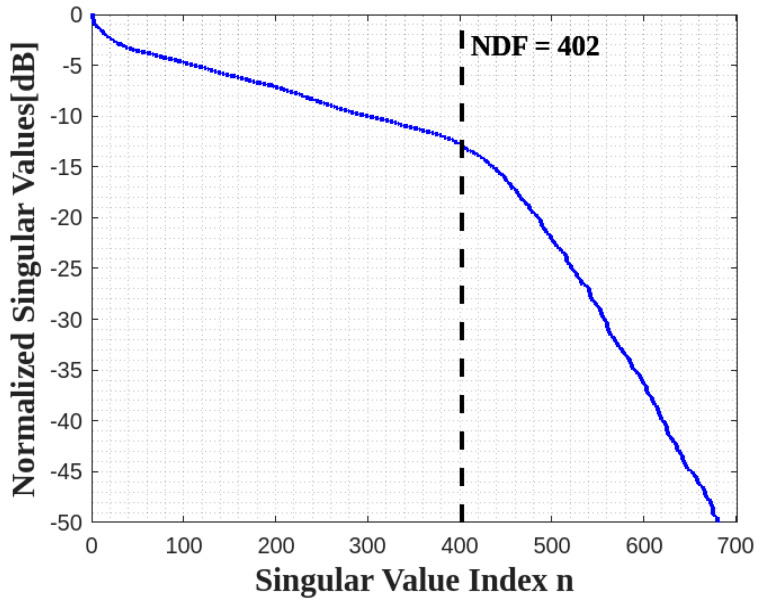
The behavior of normalized singular values for XY and XZ planes as an L shape.

In the numerical simulation, we assume that for the XY face a=b=2λ, c=2λ, and for the XZ face a=c=2λ, b=−2λ. Figure 8 confirms that the NDF is again the summation of the NDF of each face.

**Figure 9:**
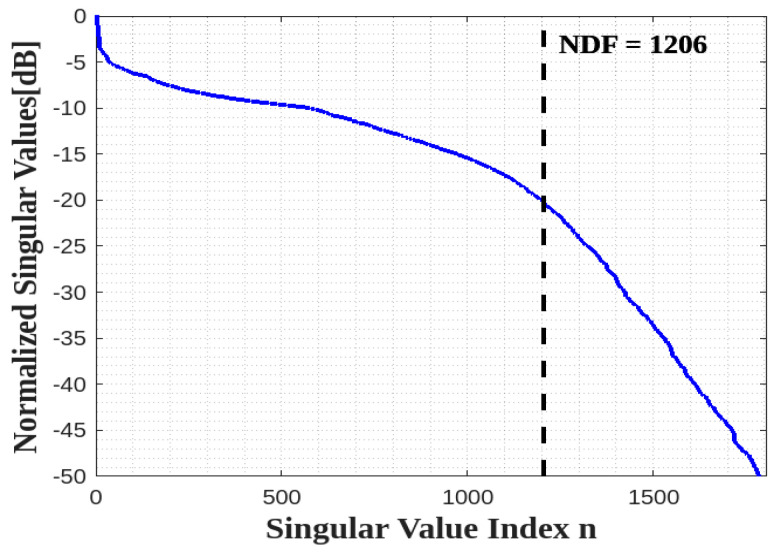
The behavior of normalized singular values for a cube ID.

In the final step, the surface cube ID is seen as the combination of its individual six faces, so that the total NDF can be estimated by considering a global matrix operator involving six separated planar domains. Accordingly, the number of the significant normalized singular values can be again expected to be connected to the geometrical features of the object. Figure 9 shows the SVs behavior for the six faces of a cube ID with a=b=c=2λ. The result confirms that the NDF of a cube is approximately equal to the summation of the NDF of six faces as expected, i.e., $NDF=32\pi \left(ab+ac+bc\right)/\lambda^{2}=4\pi S/\lambda^{2}$, if S is the measure of the surface of the parallelepiped ID.

## 4. The Optimal Number of Incident Plane Waves

A perfect reconstruction of a scatterer would require plane waves impinging from all directions and measuring scattered fields in all observation directions around the scatterer. In principle, this requires a very large number of scattering experiments by considering different angles of incidence for each impinging plane wave. Additionally, for an electrically large 3D geometry, difficulties in implementing the numerical SVD of the operator may arise because a very large matrix needs to be inverted, so increasing the execution time and requiring a powerful computer will be needed. Consequently, reducing the number of plane waves without sacrificing the reconstruction accuracy is necessary to minimize the mentioned limitations. In the following, an approach is proposed to choose the optimal number of plane waves and their directions to achieve the estimated NDF for the cube ID of the previous section, and showing that using a number of plane waves larger than the optimal one will not increase NDF, as demonstrated in [8,9] for different geometries.

**Figure 10:**
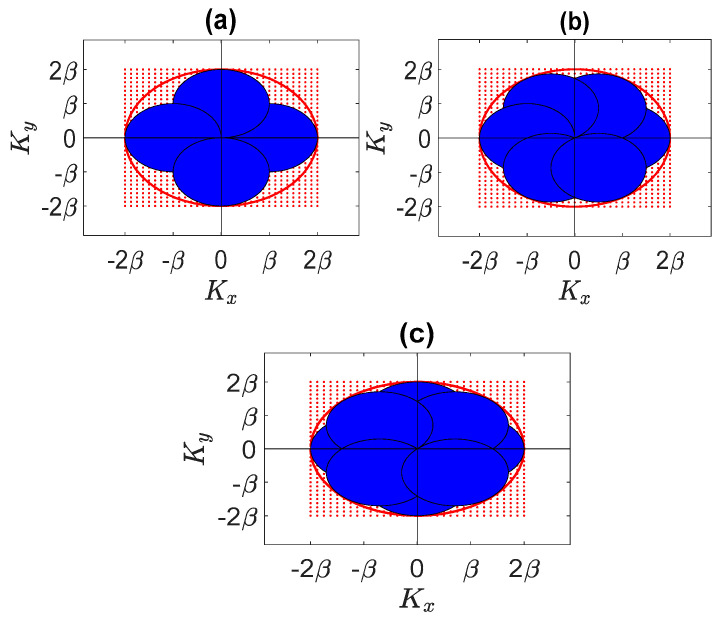
The domain spanned by (**a**) four incident plane waves, (**b**) six incident plane waves, and (**c**) eight incident plane waves in the spectral domain for a planar ID.

Let us start again with a single XY face of the cube. As mentioned in the previous section, if $k_{z}$ be 0 in (4), the 3D spectral domain shrinks to a 2D domain. When the plane waves directions ($\theta_{i},\phi_{i}$) vary continuously, a circle with a radius of 2β is spanned in all (in red in Figure 10). Accordingly, it can be expected that the same circle can be approximately filled by considering a sufficient numbers of plane waves impinging from equispaced $\phi_{i}$ directions, provided $\theta_{i}=\pi /2$. Consequently, four, six, or eight plane waves may be sufficient to cover quite fully the circle. Figure 10 shows the spectral domain area covered by four, six, and eight plane waves. As observed in Figure 10c, the circle with a radius of 2β can be approximately filled completely by eight plane waves.

Indeed, this result is independent of the shape of the planar ID and of its spatial orientation. Therefore, it can be concluded that for a planar ID a limited number of plane waves, impinging from directions belonging to the same plane, are enough to achieve the corresponding NDF in the linear inverse scattering problem.

The same geometry considered in Figure 3 of Section 3 is used in the following numerical example. In this paper, for the sake of comparison, we assume that the use of 48 impinging plane waves provides the saturation effect in the singular values computation of the scattering operator for the considered example.

**Figure 11:**
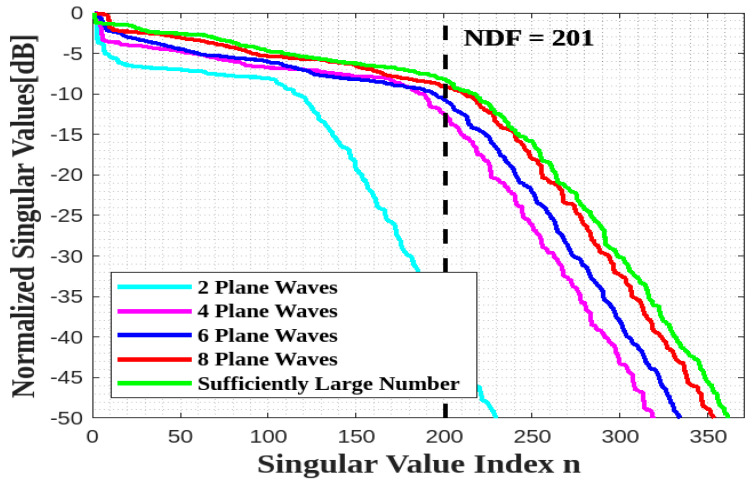
Comparison of the behavior of the normalized singular values for different numbers of impinging plane waves for a single XY face ID.

**Figure 12:**
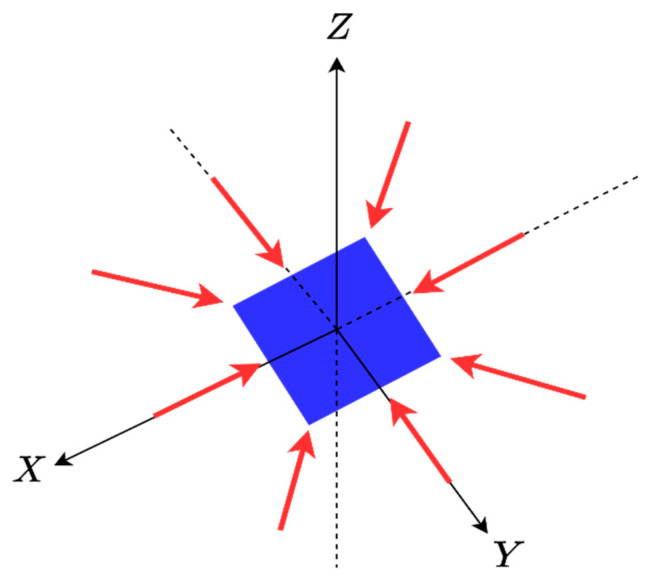
The eight directions of incident plane waves(shown as red arrows) for a XY face ID.

Figure 11 verifies that the NDF analytical estimation can be achieved by only considering a limited number of equispaced plane waves, say eight (their directions are depicted in Figure 12) for the best agreement.

**Figure 13:**
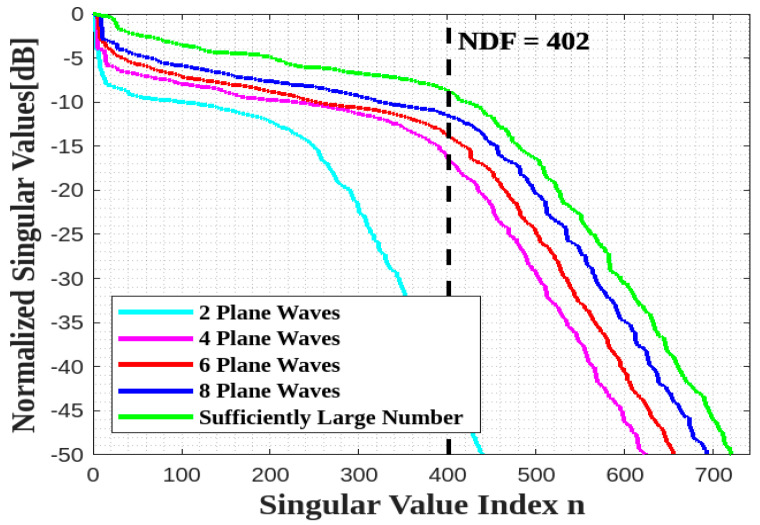
Comparison of the behavior of the normalized singular values for different numbers of impinging plane waves for two parallel XY faces.

Next, we move to the geometry of Figure 5. Since the two planar faces are parallel, it is expected that the presence of only two planar domains will not affect the previous behavior, so that the same limited number of plane waves, impinging from the same plane as above, could be enough. Figure 13 confirms that the estimated NDF is again achieved by eight plane waves.

Following that, the L-shaped ID case is considered. Now the directions of the optimal plane wave angles must be chosen by adding the ones regarding the XZ face of the cube, which are the equispaced ones whose impinging directions belong to the same plane, as follows:

(5) $$\left(\theta_{i},\phi_{i}\right)\;\;\;\;\;\;\;\;\;\;\;\;\;\;\;\;\;\;\;\;\;\;\;\;\;\;\;\;=\left(0,0\right),\;\left(\pi /4,0\right),\;\left(\pi /2,0\right),\;\left(3\pi /4,0\right),\;\left(\pi ,\pi \right),\;\left(3\pi /4,\pi \right),\;\left(\pi /2,\pi \right),\;\left(\pi /4,\pi \right)$$

When the optimal numbers of plane waves for XY face are combined with XZ face, the common angles are skipped; therefore, it results in a total number of 14 plane waves directions given by:

(6) $$\left(\theta_{i},\phi_{i}\right)=\left(\pi /2,0\right),\;\left(\pi /2,\pi /4\right),\;\left(\pi /2,\pi /2\right),\;\left(\pi /2,3\pi /4\right),\;\left(\pi /2,\pi \right),\left(\pi /2,5\pi /4\right),\;\;\;\left(\pi /2,3\pi /2\right),\;\left(\pi /2,7\pi /4\right),\left(0,0\right),\;\left(\pi /4,0\right),\;\left(3\pi /4,0\right),\;\left(\pi ,\pi \right),\;\left(3\pi /4,\pi \right),\;\left(\pi /4,\pi \right)$$

**Figure 14:**
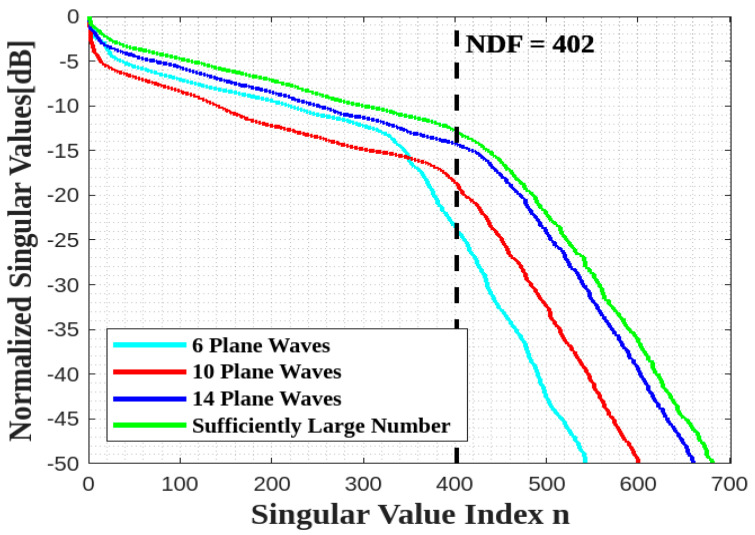
Comparison of the behavior of the normalized singular values for different numbers of impinging plane waves for the L shape ID.

As can be seen by the numerical result of Figure 14, 14 incident plane waves are sufficient to obtain the estimated NDF.

Finally, starting from the above two latter results to introduce the optimal number of plane waves and their directions for all sides of a cube, it needs to start by considering (7) and to add as the optimal plane wave angles for the YZ face of the cube.

(7) $$\left(\theta_{i},\phi_{i}\right)=\left(0,\pi /2\right),\;\left(\pi /2,\pi /2\right),\;\left(\pi ,\pi /2\right),\;\left(0,3\pi /2\right),\;\;\left(\pi /2,3\pi /2\right),\;\left(\pi ,3\pi /2\right),\;\left(\pi /4,\pi /2\right),\;\left(3\pi /4,3\pi /2\right)$$

When (7) is combined with (8), and the common angles are skipped, 20 plane waves are obtained, whose directions are:

(8) $$\left(\theta_{i},\phi_{i}\right)=\left(\pi /2,0\right),\;\left(\pi /2,\pi /4\right),\;\left(\pi /2,\pi /2\right),\;\left(\pi /2,3\pi /4\right),\;\left(\pi /2,\pi \right),\;\;\left(\pi /2,5\pi /4\right),\;\left(\pi /2,3\pi /2\right),\;\left(\pi /2,7\pi /4\right),\left(0,0\right),\;\left(\pi /4,0\right),\;\left(3\pi /4,0\right),\;\left(\pi ,\pi \right),\;\;\left(3\pi /4,\pi \right),\;\left(\pi /4,\pi \right),\;\left(0,\pi /2\right),\left(\pi ,\pi /2\right),\;\left(0,3\pi /2\right),\;\left(\pi ,3\pi /2\right),\;\left(\pi /4,\pi /2\right),\;\left(3\pi /4,3\pi /2\right)$$

**Figure 15:**
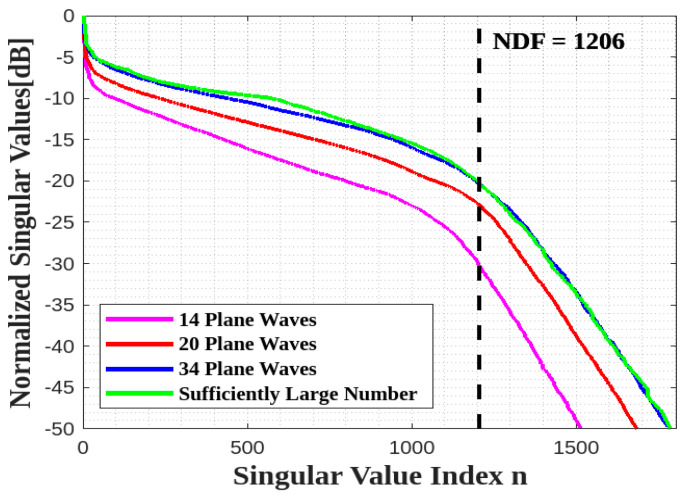
Comparison of the behavior of the normalized singular values for different numbers of impinging plane waves for all sides of a cube ID.

Figure 15 confirms that the whole estimated NDF for a parallelepiped surface ID can be achieved by only 20 plane waves, and considering more does not affect the NDF estimation.

## 5. Application to Microwave Tomography of Dielectric Object

**Figure 16:**
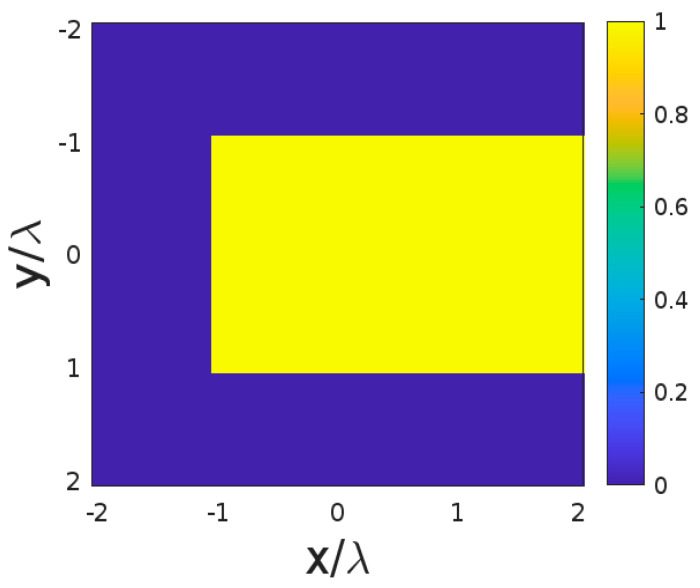
The geometry of the dielectric object in ID.

In this section, an application in microwave tomography of dielectric object is introduced to show usefulness of the theoretical discussion. The surface of the XY face of a cube is considered for this application. As shown in Figure 16, the range of the ID in the x and y axes is from −2λ to 2λ. We assume that a dielectric object with a size of 3λ×2λ is located within the ID in free space. The background medium is air, and the contrast between the dielectric object and the background is 1. 

In this application, the considered object is reconstructed under three different scenarios which vary in terms of the number and direction of impinging plane waves. The first scenario involves reconstructing the object using a sufficiently large number of incident plane waves from all directions. In the second scenario, reconstruction is implemented using only eight correct directions of plane waves belonging to the XY plane, the same as the ID (as shown in the previous section). Finally, the third scenario uses eight wrong angles of plane waves impinging from the XZ plane. The TSVD inversion method is used for all scenarios, and the truncation level must be chosen in each case according to the discussion of the previous Section. 

**Figure 17:**
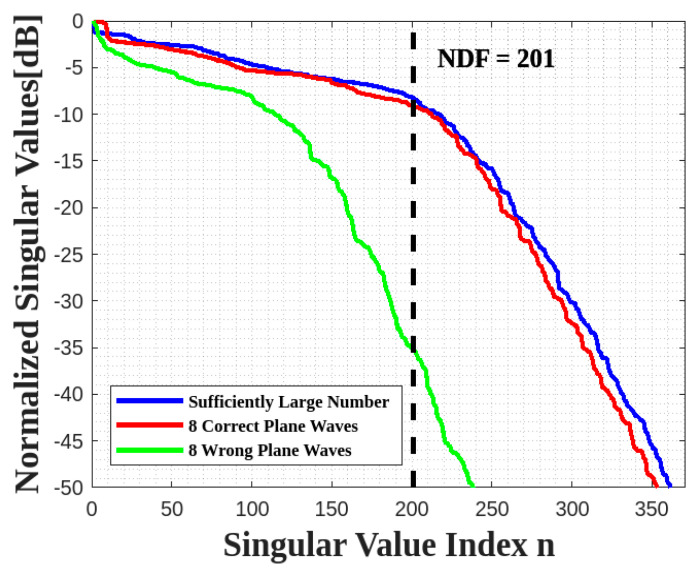
Comparison of the behavior of the normalized singular values for three scenarios for a XY face.

The behaviors of the normalized singular values for all scenarios are plotted in Figure 17 to compute their NDF, which is then used as the truncation level. The estimated NDF is achieved in the first and second scenarios, whereas only half of the estimated NDF is obtained in the third scenario.

The Relative Root Mean Square (RRMS) criterion, that is the Euclidean norm of the difference between the reconstructed images and the reference one of Figure 16 over the norm of the reference image, is applied to compare the different reconstructions. It was chosen because the TSVD provides the best results under that norm.

**Figure 18:**
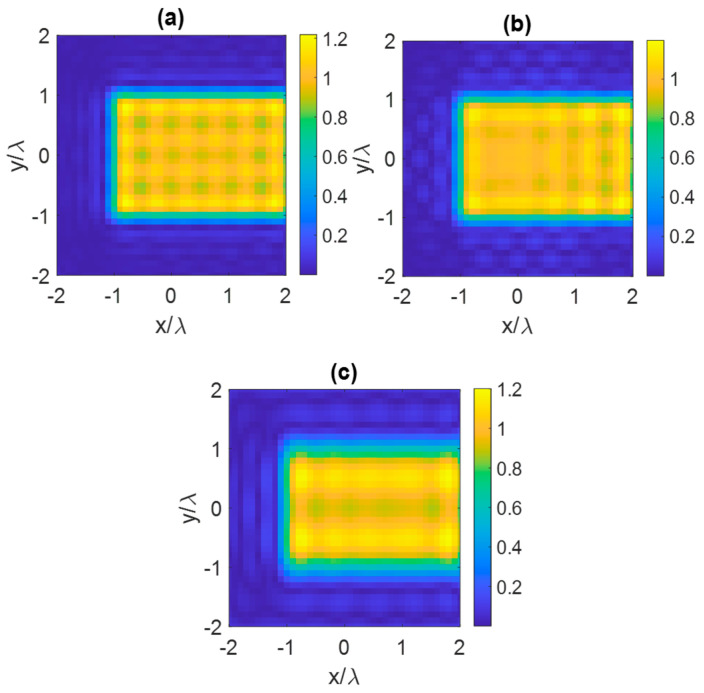
Reconstructed images of a dielectric the scatterer within a planar ID for three scenarios from: (**a**) sufficiently large of impinging plane waves, (**b**) plane waves belonging to the same plane as the object, and (**c**) form eight plane waves belonging to a different plane.

Figure 18 displays the reconstructed images for all scenarios. The RRMS values for the three scenarios are 0.17, 0.19, and 0.24, respectively. The RRMS of the reconstruction for the second scenario is close to the first one, whereas the RRMS for the third reconstruction is larger than the others. As shown in Figure 18a,b, the object can be reconstructed almost perfectly, whereas in the other cases the borders of the object appear more defocused (as seen in Figure 18c), indicating a lower reconstructed slope. Moreover, the reconstructed object in Figure 18c appears blurry, indicating that incorrect directions of plane waves can result in a lower NDF and negatively impact the result of the imaging algorithm.

## 6. Conclusions

The NDF evaluation of the far field scattered by 3D geometry has been considered in the 3D domain. The surface of a cube has been addressed for investigation since a theoretical evaluation is possible. The results have shown that its NDF is approximately equal to the summation of the NDF of the six sides. To reduce the computational effort of an SVD-based imaging algorithm, which involves matrix inversion, we investigated the optimal number of plane waves and their directions needed to achieve the estimated NDF. In this way, not only the size of the matrix to be inverted, but also the time to collect data in experimental setups(i.e., the required number of transmitters) can be reduced. This goal has been achieved with the same step by step procedure employed for the NDF evaluation, examining for one side, two sides, and six sides of the cube in order. Finally, we demonstrated how the NDF, number of plane waves, and their directions can affect the reconstruction result in microwave imaging.

## Appendix A

In this appendix, we recall the mathematical approach used in [7,8,9] to estimate the total NDF of a collection of linear sources/scatterers in linear inverse source and scattering problems. Let us start with two separated sources/scatterers. The individual radiated/scattered fields are defined by the operators $E_{1}=T_{1}\left(S_{1}\right)$ and $E_{2}=T_{2}\left(S_{2}\right)$. The relevant operator of the total field can be considered as the sum of the contributions of two sources/scatterers:

(A1) $$E=T_{1}\left(S_{1}\right)+T_{2}\left(S_{2}\right)=[T_{1}\;\;\;\;\;\;T_{2}]\;\left[\begin{array}{c} S_{1}\\ S_{2} \end{array}\right]=T\;S$$

which can be recast under a matrix formulation.

The corresponding adjoint operator of T is defined as:

(A2) $$T^{+}=\left[\begin{array}{c} T_{1}^{+}\\ T_{2}^{+} \end{array}\right]$$

For the total SVs estimation, instead of discussing the T operator, we can equivalently consider the composite operator $T^{+}T$, which provides the matrix operator:

(A3) $$\left(T^{+}T\;\right)\left(S\right)=\left[\begin{array}{cc} T_{1}^{+}T_{1} & T_{1}^{+}T_{2}\\ T_{2}^{+}T_{1} & T_{2}^{+}T_{2} \end{array}\right]$$

Then, the norm of off-diagonal terms can be considered, which is less than the norm of the pertinent kernel functions. It results that when two sources/scatterers are sufficiently far apart, the operator norms are expected larger for the diagonal contributions than for the off-diagonal terms. Thus, it results in a diagonal block operator:

(A4) $$\left(T^{+}T\;\right)\left(S\right)≅\left[\begin{array}{cc} T_{1}^{+}T_{1} & 0\\ 0 & T_{2}^{+}T_{2} \end{array}\right]$$

This means that the total SVs are the combination of the SVs of each individual operator. Finally, denoting the NDF of the radiated/scattered fields of each source/scatterer by $NDF_{1}$ and $NDF_{2}$, respectively, the NDF of the configuration can be given by:

(A5) $$NDF_{T}≅NDF_{1}+NDF_{2}$$

The whole discussion can be extended to more than two sources/scatterers straightforwardly.
